# Improvement After Vestibular Rehabilitation Not Explained by Improved Passive VOR Gain

**DOI:** 10.3389/fneur.2020.00079

**Published:** 2020-02-20

**Authors:** Jennifer L. Millar, Yoav Gimmon, Dale Roberts, Michael C. Schubert

**Affiliations:** ^1^Department of Physical Medicine and Rehabilitation, Johns Hopkins University School of Medicine, Baltimore, MD, United States; ^2^Laboratory of Vestibular NeuroAdaptation, Department of Otolaryngology - Head and Neck Surgery, Baltimore, MD, United States; ^3^Department of Physical Therapy, Faculty of Health Sciences, Ben-Gurion University of the Negev, Beer-Sheva, Israel; ^4^Department of Neurology, Johns Hopkins University School of Medicine, Baltimore, MD, United States

**Keywords:** vestibular rehabilitation, dynamic visual acuity, vestibulo-ocular reflex gain, compensatory saccades, otolith function

## Abstract

Gaze stability exercises are a critical component of vestibular rehabilitation for individuals with vestibular hypofunction and many studies reveal the rehabilitation improves functional performance. However, few studies have examined the vestibular physiologic mechanisms (semicircular canal; otolith) responsible for such recovery after patients with vestibular hypofunction complete gaze and gait stability exercises. The purpose of this study was to compare behavioral outcome measures (i.e., visual acuity during head rotation) with physiological measures (i.e., gain of the vestibulo-ocular reflex) of gaze stability following a progressive vestibular rehabilitation program in patients following unilateral vestibular deafferentation surgery (UVD). We recruited *n* = 43 patients (*n* = 18 female, mean 52 ± 13 years, range 23–80 years) after unilateral deafferentation from vestibular schwannoma; *n* = 38 (25 female, mean 46.9 ± 15.9 years, range 22–77 years) age-matched healthy controls for dynamic visual acuity testing, and another *n* = 28 (14 female, age 45 ± 17, range 20–77 years) healthy controls for video head impulse testing. Data presented is from *n* = 19 patients (14 female, mean 48.9 ± 14.7 years) with UVD who completed a baseline assessment ~6 weeks after surgery, 5 weeks of vestibular physical therapy and a final measurement. As a group, subjective and fall risk measures improved with a meaningful clinical relevance. Dynamic visual acuity (DVA) during active head rotation improved [mean ipsilesional 38.57% ± 26.32 (*n* = 15/19)]; mean contralesional 39.96% ± 22.62 (*n* = 12/19), though not uniformly. However, as a group passive yaw VOR gain (mean ipsilesional pre 0.44 ± 0.18 vs. post 0.44 ± 0.15; mean contralesional pre 0.81 ± 0.19 vs. post 0.85 ± 0.09) did not show any change (*p* ≥ 0.4) after rehabilitation. The velocity of the overt compensatory saccades during ipsilesional head impulses were reduced after rehabilitation; no other metric of oculomotor function changed (*p* ≥ 0.4). Preserved utricular function was correlated with improved yaw DVA and preserved saccular function was correlated with improved pitch DVA. Our results suggest that 5 weeks of vestibular rehabilitation using gaze and gait stability exercises improves both subjective and behavioral performance despite absent change in VOR gain in a majority of patients, and that residual otolith function appears correlated with such change.

## Introduction

Gaze stability refers to the eyes maintaining a stable position in space (and the head in this context) relative to a head movement, which is essential for providing stable visual acuity during walking and other activities of daily living. While walking, healthy controls experience gait velocity ranging from 0.6 to 2.5 m/s while the head translates in frequencies ranging from 1.4 to 2.5 Hz (1, 2). When running, the frequency of head rotation in pitch is twice that of yaw (pitch median = 3.2 Hz, yaw median <2 Hz) and can reach peaks from 15 to 20 Hz (2). Given the high range of velocity and frequency of head motion encountered during such typical life, healthy vestibular function is essential to ensure gaze stability (3). When lesioned, the vestibulo-ocular reflex (VOR) is unable to stabilize the eyes during head motion and visual acuity degrades (4–6).

Gaze stability exercises are considered a critical component of vestibular rehabilitation for individuals with vestibular hypofunction (7). Prior studies have shown gaze stability exercises are effective at improving visual acuity during active head rotation (dynamic visual acuity) as well as postural stability in patients with unilateral vestibular hypofunction (UVH) (8, 9) and following vestibular schwannoma resection (10). Interestingly, patient's self-report of oscillopsia post intervention does not correlate with improved dynamic visual acuity (DVA), nor does age, time from onset, initial DVA score, duration or type of exercise (8, 11).

There is evidence that the lesioned VOR gain (eye/head velocity) to slow velocity passive head rotation can be improved. Enticott et al. (5), reported patients who performed gaze stabilization exercises following vestibular schwannoma tumor resection did reduce their asymmetry of VOR gain, as measured during slow velocity (60°/s) rotatory chair testing. Additionally, those patients reported reduced dizziness compared to control subjects. More recently, Sadeghi et al. have used passive ipsilesional whole-body rotation to reduce VOR asymmetry (12). There is some evidence the VOR gain can improve to faster, active (self-generated) head velocity rotation as well. Measuring the eye and active head velocity (scleral search coil) before and after a 5 weeks of vestibular rehabilitation in patients with unilateral vestibular hypofunction due to presumed vestibular neuritis, Schubert et al. (9) described a 35% improvement in ipsilesional VOR gain during the active DVA testing [mean gain 0.7 ± 0.2 to 0.9 ± 0.2 (*p* < 0.05)]. Additionally, the patient subjects recruited a larger number of compensatory saccades (saccades in the direction of the deficient VOR) to assist with gaze stability that was dependent on magnitude of the VOR gain (9, 13). Others have shown a similar inverse relationship with the presence of compensatory saccades and magnitude of VOR gain change after vestibular rehabilitation (14).

To our knowledge, no study has assessed the effect of active head rotation gaze stability exercises on functional and physiological outcome measures of vestibular function. The purpose of this study was to compare behavioral (i.e., DVA) and functional (i.e., fall risk) outcome measures with vestibular physiological measures including semicircular canal (i.e., VOR gain) and otolith (saccule and utricule) function following a progressive 5 week vestibular rehabilitation program in patients following unilateral vestibular deafferentation (UVD) surgery.

## Methods

### Subjects

We recruited *n* = 43 patients (*n* = 18 female, mean 52 ± 13 years, range 23–80 years) post UVD surgery due to vestibular schwannoma tumor resection; 19 of those patients completed the study (14 female, mean 48.9 ± 14.7 years). We also recruited and collected data in *n* = 38 (*n* = 25 female, mean 46.9 ± 15.9 years, range 22–77 years) aged matched healthy controls for DVA testing, and another *n* = 28 (14 female, age 45 ± 17, range 20–77 years) healthy controls for video head impulse testing. Patients were excluded for traumatic brain injury, cerebrovascular accident, or multiple sclerosis. The study was approved by the Johns Hopkins University Institutional Review Board and written informed consent was obtained from each individual.

Data presented below is from the *n* = 19 patients with UVD who completed an initial measurement, 5 weeks of vestibular physical therapy (VPT), and a final measurement. Sixteen patients were lost to follow up given they resided out of state; the final eight patients were excluded from the data analysis due to extended time between surgery and initial testing or extended time between pre and post VPT testing.

### Overview

The pre VPT measure was collected ~6 weeks post vestibular schwannoma tumor resection in an outpatient clinic setting (39 ± 31 days). The post VPT measure was collected mean 56 ± 25 days from the pre VPT measure. Outcome measures were collected from the physiologic (i.e., VOR gain), performance (i.e., computerized DVA), and subjective (i.e., dizziness handicap inventory) domains. Data collection and intervention was performed by one of two research physical therapists (JLM, YG). VPT included 5 weeks of gaze stability exercises as well as static and dynamic postural stability tasks (8). Each patient was given a home exercise program and followed up weekly with in-clinic outpatient visits. Each patient received 5 weeks of treatment. All data was stored into a customized online cloud database (REDCap Vanderbilt University) for offline analysis.

### Physiologic Measures

#### The Video Head Impulse Test (vHIT)

The vHIT (ICS Otometrics, Natus Medical Incorporated, Denmark) measured VOR gain (eye velocity/head velocity) as well as metrics of the compensatory saccades [latency, frequency, velocity, and the overall PR score (measure of variability in latency, termed as gathered or scattered)]. Compensatory saccades are defined as those saccades occurring within 350 ms of the onset head rotation, in the direction of the deficient VOR. Covert saccades occur during the head rotation, overt saccades occur after the head rotation ends. VOR gain values within 0.8–1.2 with standard deviation <0.12 were considered normal (15, 16).

Patients were seated 1 meter from a stationary visual target, in room light. Right eye velocity and head velocity were sampled at 220 Hz in response to passive right and left head rotations. Care was taken to avoid the examiner's hands making contact with the head strap to avoid goggle slip. At least 12 passive head rotations were performed in three planes: yaw, right anterior/left posterior (RALP) and left anterior/right posterior (LARP). vHIT traces were deleted if the eye velocity trace preceded head velocity, or if the passive head rotation trace did not match the acceleration profile suggested by the manufacturer.

#### Vestibular Evoked Myogenic Potential Test (VEMP)

Both ocular and cervical VEMP was measured using the Otometrics VEMP Chartr EP 200 System (Natus Medical Incorporated, Denmark). A burst tone stimulus [loud clicks, typically 95–105 decibels above normal hearing level (dB nHL), in 200 ms intervals] was applied during both ocular and cervical (O and C VEMP) paradigms. VEMP testing was considered abnormal for reduced sound threshold (dB) and/or latency of the positive and negative response being greater than the mean and 2SD above age matched controls (17). Percent asymmetry ratio was calculated for the ocular and cervical VEMP tone burst stimulus:

$$\begin{array}{l} \text{Asymmetry ratio}(\text{AR})=\\ \;100\%\frac{\text{X}\left(\text{Left amplitude }-\text{ Right amplitude}\right)}{\left(\text{Left amplitude }+\text{ Right amplitude}\right)} \end{array}$$

### Subjective Measures

#### Dizziness Handicap Inventory (DHI)

Patients reported their perceived level of disability via the DHI. The DHI is a 25-item subjective measure that collects data on how disabling the patients perceive their dizziness is affecting them. Clinically relevant change scores were defined as a decrease in the DHI of either 18 points or 42% from the pre-treatment level (18, 19).

#### Activities-Specific Balance Confidence Scale (ABC)

The ABC evaluates a subject's level of perceived balance confidence by asking them to rate confidence performing various daily activities from 0 (no confidence) to 100% (complete confidence). Total scores >80% are interpreted as having a high level of balance confidence and scores below 67% predict a person is at risk for falls (20). The ABC has excellent test-retest reliability (*r* = 0.92) (21, 22).

### Performance Measures

#### Dynamic Visual Acuity (DVA)

We developed a custom, portable computerized DVA test using a Samsung Galaxy Pro tablet (Seoul, South Korea) with a single inertia measurement unit (XSENS Technologies, Enschede, Netherlands) mounted on a headband. Visual acuity was first measured during head still and then during active horizontal and vertical sinusoidal head rotation (right, left, up, down) while the subject sat 200 cm from the tablet. A minimum of >120°/s of active head rotation was required to generate the random optotype presentation, with no maximum head velocity limitation. Ten individual optotypes (capital letters C D H K N O S R V Z) were presented and scores were tabulated in the logarithm of the minimal angle resolution (LogMAR). Possible LogMAR scores ranged from −0.3 to 1.7 (Snellen equivalent of 20/10 to 20/800). Details of the DVA paradigm, as well as normative values can be found at Li et al. (23).

#### Dynamic Gait Index (DGI)

The DGI is an 8-item functional outcome measure that asks subjects to perform various dynamic gait tasks (i.e., walk and then turn 180°, walk and step over an obstacle). The DGI measures fall risk with scores <19/24 points reflecting a 2.58 times greater likelihood to have fallen in the previous 6 months (24). The DGI has excellent inter-rater (*r* = 0.96) and intra-rater (*r* = 0.98) reliability in older adults (25). A change score of > 3 is considered clinically significant (26).

#### Timed Up and Go (TUG)

The TUG measures the duration to stand, walk 3 m, and turn 180° before returning to sit. The TUG indicates fall risk when scores are >13.5 s in older adults with vestibular disorders (27). The TUG has excellent inter and intra-rater reliability (28).

#### Gait Speed and Endurance

The Ten Meter Walk Test (10MWT) tasks subjects to walk 10M during which their preferred gait speed is determined. The 2 min walk test (2MWT) tasks subjects to walk for 2 min while distance (endurance) is measured. The minimally clinical important difference (MCID) for gait speed is dependent on patient population, and not explicitly known for patients with vestibular disorders. We selected a substantial meaningful change at 0.1 m/s (29, 30). The 2MWT has excellent reliability (*r* = 0.95) (30, 31) and the minimal detectable change (MDC) is 12.2 m (31).

### Exercise Group Categorization

Patients were placed in an exercise category (A, B, or C) to ensure high intensity, yet safe training to achieve maximum benefits of the VPT program. This categorization served a second purpose of limiting variability of the exercises prescribed. Placement into one of the three groups was based on the combined results of an individual's gait speed and scores on the DHI, ABC, TUG, and DGI (Table 1). Scores were tallied with patients being placed into either A, B, or C categories. In the case of an equivalent score between individual sub-scores (i.e., a patients' ABC score met the criteria for the B subcategorization, yet the same patients' DGI criteria placed them in C categorization), exercises from the more challenging group (group C in this example) were prescribed.

**Table 1:** ABC treatment categorization.

**VPT difficulty**	DHI^*^	**ABC^**&**^**	**TUG^**∧**^**	**DGI^**#**^**	**Gait ^**%**^** **< age 70**	**Gait ^**%**^** **>age 70**
A	>60	≤ 30	>14	<15	0.8	0.7
B	31–60	31–65	11–14	15–18	1.1	1.0
C	≤ 30	>65	<11	≥19	1.4m/s	1.3m/s

*A, Least Challenge; B, Moderate Challenge; C, Most challenge; VPT, vestibular physical therapy; DHI, Dizziness Handicap Inventory; TUG, Timed Up and Go, DGI, Dynamic Gait Index*.

* Jacobson and Newman (18) and Whitney et al. (32);

& Lajoire and Gallagher (20) and Whitney et al. (33);

∧ Whitney et al. (24);

# Whitney et al. (24);

% *Bohannon (34), and Bohannon and Glenney (35); ^%^Perera et al. (29); ^%^van Loo et al. (36)*.

### Vestibular Rehabilitation Program

Each exercise group (A, B, C) completed 6 exercises including 2 active gaze stability, 2 static balance, and 2 dynamic balance exercises (please see Data Sheet 1). Patients were asked to perform each exercise for 3 repetitions of 1.5 min duration each for a total of 27 min, 7 days per week for 5 weeks (7). Additionally, patients were instructed to complete a daily walk. Gaze stability exercises included active sinusoidal head rotations at fast head velocities with the understanding that the visual target should appear stable and clear. Each week, a progressively more difficult set of gaze stability, static, and dynamic balance exercises were provided (i.e., gaze stability exercise done against a busy visual background), and provided to the patient that included detailed verbal, written, and illustrated instructions. The study team monitored the patients' ability to perform the exercises appropriately in a clinic setting, when possible, and also monitored the patient compliance based on the subjects' self-completed exercise flow sheets. Some patients opted to participate with outpatient VPT close to their home (in addition to our exercise program). Those patients (*n* = 3) who additionally participated in an outpatient VPT program agreed to complete only the research study-prescribed home exercise program.

### Statistical Analysis

Statistical analysis was performed using SPSS (version 26, Chicago, IL, USA) software. All variables were normally distributed, thus parametric analysis was performed. A paired *t*-test was performed to compare variables between pre and post VPT. The level of statistical significance was set at alpha ≤0.05. As sample size permitted, simple correlations were determined in Excel using the Correl function (MS office, Redmond WA, USA). In addition to statistical significance, a change score of 10% in the compensatory saccade physiologic metrics and dynamic visual acuity was considered improved. For VOR gain, a change in magnitude > 0.06 was considered significant (37).

## Results

### Vestibular Physiological Outcomes

#### vHIT

**Figure 1:**
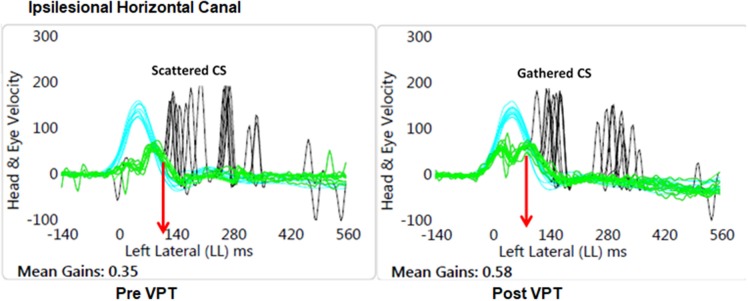
Graphical plot of the improved VOR gain and reduced variability of the latency of the compensatory saccades (CS). Also, note the CS latency (red arrow) has reduced at the Post VPT plot. Blue, head velocity; Green, eye velocity; Black, compensatory saccades; VPT, vestibular physical therapy.

As expected, all patients had reduced VOR gain during passive ipsilesional yaw head impulse testing (Table 2). The average passive head velocity during vHIT for yaw rotations was 177.47 ± 55.7°/s. Although three subjects showed improved (>0.06) ipsilesional VOR gain to passive head impulse testing (mean 59 ± 14.6%), as a group yaw VOR gain (vHIT) did not show significant change after VPT (Table 2). Velocity of the overt compensatory saccades (CS) during ipsilesional head impulses were significantly reduced after VPT (mean 18 ± 6.6%), but no other metric of the CS showed any group changes (*p* ≥ 0.4, Table 2). Nine subjects did show reduction (>10%) in latency variability of the CS (PR score, mean 45 ± 19%), though this was not statistically significant (Figure 1).

**Table 2:** Physiologic measures of change in vHIT gain and compensatory saccades (mean + 1 SD).

**Oculomotor function**	**Pre**	**Post**	***p*-value (2-tailed)**
Control left yaw	0.93 ± 0.05		
Control right yaw	0.99 ± 0.05		
Contralesional yaw	0.81 ± 0.19	0.85 ± 0.09	0.412
Ipsilesional yaw	0.44 ± 0.18	0.44 ± 0.15	0.984
Yaw % asymmetry	50.16 ± 19.76	50.95 ± 15.01	0.813
Contralesional anterior canal	0.68 ± 0.19	0.68 ± 0.18	0.989
Ipsilesional anterior canal	0.43 ± 0.26	0.39 ± 0.21	0.331
Contralesional posterior canal	0.81 ± 0.21	0.86 ± 0.22	0.229
Ipsilesional posterior canal	0.45 ± 0.14	0.50 ± 0.28	0.505
Variability of latency of saccades (PR ipsilesional score)	58.69 ± 30.12	48.94 ± 23.31	0.280
Ipsi covert saccade latency (ms)	124.57 ± 28.2	115.79 ± 39.4	0.485
Ipsi covert saccade velocity (°/s)	217.79 ± 71.2	220.64 ± 54.2	0.895
Ipsi overt saccade latency (ms)	211.06 ± 39.4	211.00 ± 31.5	0.996
**Ipsi overt saccade velocity (°/s)**	217.75 ± 63.7	200.81 ± 57.5	0.042^*^

*vHIT, video head impulse test; ms, milliseconds; ipsi, direction of head rotation toward the lesioned side; covert, compensatory saccade during head rotation; overt, compensatory saccades after head rotation; PR, range of variability in the latency of compensatory saccades. A low PR score reflects maximum gathered responses vs. a high PR score reflects maximum scattered responses*.

* *Denotes significance at p < 0.05*.

#### VEMP

Six of our 19 subjects did not have complete VEMP data collection due to equipment failure or the external auditory meatus being sewn closed (*n* = 1). Four of the remaining 13 subjects had absent and/or >2SD of mean cVEMP asymmetry ratios (mean 88 ± 16%). Five of the 13 subjects had absent and/or > 2SD oVEMP asymmetry ratios (mean 93 ± 9%). The rest of the subjects had measurable ocular (mean 26 ± 18%) or cervical (mean 35 ± 14%) VEMP responses within two SD of healthy controls (17).

### Subjective Measures

As a group, the dizziness handicap inventory (DHI) total score as well as each subscale significantly improved (Table 3). The ABC scale also showed significant improvement after VPT. Neither age nor exercise compliance were correlated with the change in the DHI or the ABC.

**Table 3:** Change in subjective and performance outcome measures.

	**ABC (%)**	**DHI-P**	**DHI –F**	**DHI -E**	**DHI -total**	**DGI**	**TUG (sec)**	**10MWT (m/s)**	**2MWT (m)**
Pre	67.3 ± 21.0	15.2 ± 6.8	12.7 ± 9.6	20.8 ± 9.7	48.7 ± 23	20.7 ± 4.3	8.5 ± 1.8	1.3 ± 0.3	160.3 ± 27.6
Post	87.1 ± 12.7	10.4 ± 7.7	8.0 ± 8.6	10.2 ± 11	26.9 ± 25	23.2 ± 1.7	7.5 ± 1.0	1.4 ± 0.2	164.1 ± 46.1
% Change	29%^*^ P = 0.002	32%^*^ P = 0.002	37%^*^ p = 0.00	51%^*^ p = 0.000	45%^**^ p = 0.000	12%^**^ p = 0.016	12%^*^ p = 0.002	8%^**^ p = 0.009	2%

*ABC, Activities Balance Confidence Scale; DHI, Dizziness Handicap Inventory; P, Physical DHI; F, Functional DHI; E, Emotional DHI; DGI, Dynamic Gait Index; TUG, Timed Up and Go test; 10MWT, gait velocity: 2MWT, gait endurance*.

** Minimal Clinically Important Difference;

* *Statistically significant*.

### Performance Measures

The DGI, TUG, and gait speed all improved after VPT (Table 3). Additionally, improvement in DGI was negatively correlated with tumor size (*r* = −0.4). There was no correlation between age and exercise compliance and the change score for the ABC, DGI, TUG, or gait speed.

#### Dynamic Visual Acuity

**Figure 2:**
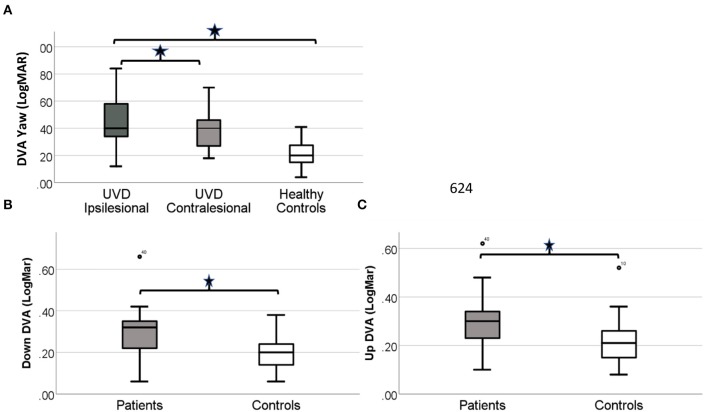
Simple boxplot of the DVA scores for healthy controls and patients with UVD before VPT. **(A)** DVA scores for the patients with UVD are significantly worse for both ispi and contra-lesional active head rotation (*p* < 0.0001). DVA scores for the patients with UVD are significantly worse (*p* < 0.01) for up **(B)** and down **(C)** active head rotation. The thick line in the middle is the median. The top and bottom box lines show the first and third quartiles. The whiskers show the maximum and minimum values. Outliers are noted by the circles.

DVA scores for the healthy controls were similar (*p* = 0.64) in yaw for left (mean 0.21 ± 0.11 LogMAR) and right (0.20 ± 0.9 LogMAR) active head rotation and thus were combined and compared against the patients with UVD. DVA for the patients with UVD was worse than the healthy controls for yaw [ipsilesional (*p* < 0.001); contralesional (*p* < 0.001)] and pitch [up (*p* = 0.006); down (*p* = 0.003)] active head rotation (Figure 2).

**Figure 3:**
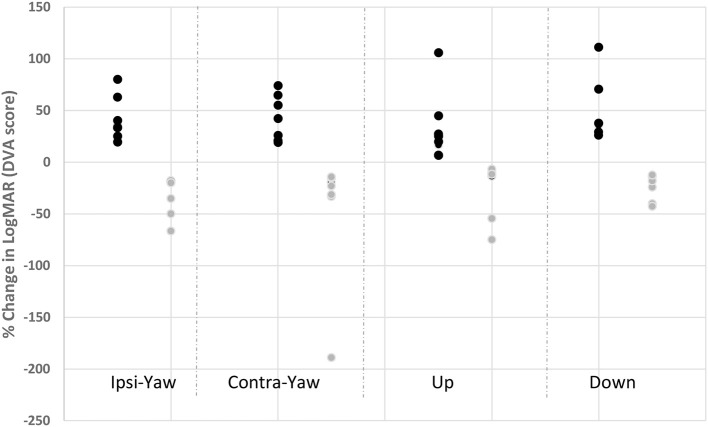
Change in individual DVA scores for head rotation in responders and non-responders. Dark circles represent those subjects with improved DVA score after vestibular rehabilitation (responders); gray circles represent those subjects with worse DVA score after vestibular rehabilitation. Ipsi yaw, ipsilesional head rotation in yaw; Contra yaw, contralesional head rotation in yaw; Up, upward head rotation; Down, downward head rotation.

As a group, DVA did not improve (*p* ≥ 0.13) for any head direction (Table 4). However, 79% of our subjects (*n* = 15/19) did show improved DVA by at least 10%. A within-subject sub-analysis was performed on those patients who showed a minimum 10% improvement vs. those that did not. Ten percent was chosen as this represents the difference in LogMAR between lines of visual acuity (i.e., LogMAR 0.0 vs. 0.1). Within the positive responders, the mean improvement in DVA during ipsilesional head rotation was significant at 38.57% ± 26.32 (*n* = 15/19), and 39.96% ± 22.62 (*n* = 12/19) for contralesional yaw head rotation (*p* < 0.001). The magnitude of improved DVA score was negatively correlated (*r* = −0.37) with the magnitude of residual ocular VEMP function. The cervical VEMP response was not correlated with any change in yaw DVA (*r* = 0.1). For pitch down, DVA improved 59.23 ± 47.47% (*n* = 14/19, *p* < 0.01), which was correlated with the residual magnitude of both oVEMP (*r* = −0.53) and cVEMP (*r* = −0.33) asymmetry ratios. For pitch up, DVA improved 34.29 ± 112.76% (*n* = 3/19, *p* = 0.03; Figure 3). Correlations were not done between VEMP response and DVA up due to limited sample.

**Table 4:** Dynamic visual acuity scores for active head rotation in yaw and pitch.

**UVD *n* = 19**	**Static**	**Ipsi**	**Contra**	**Up**	**Down**
Pre	0.01 ± 0.18	0.45 ± 0.22	0.38 ± 0.21	0.31 ± 0.21	0.31 ± 0.19
Post	0.02 ± 0.29	0.40 ± 0.38	0.33 ± 0.26	0.27 ± 0.3	0.28 ± 0.27
	**Static**	**Left**	**Right**	**Up**	**Down**
Healthy controls *n* = 38	−0.06 ± 0.19	0.15 ± 0.22	0.15 ± 0.23	0.13 ± 0.23	0.15 ± 0.22

*UVD, unilateral vestibular deafferentation; LogMAR, logarithm of the minimal angle of resolution. ipsi, direction of head rotation toward the lesioned side; contra, direction of head rotation toward the contralesional side. A LogMAR score of 0 equates with 20/20 visual acuity on the Snellen acuity scale. A lower LogMAR scores reflect better visual acuity*.

Of the negative responders, the mean reduction in % DVA change for ipsilesional head rotation was −122.76 ± 11.55 (*n* = 4/19 patients did not improve) and −70.38 ± 62.19 (*n* = 7/19 patients did not improve) during contralesional head rotation. Vertical DVA worsened by −60.89 ± 76.41% (*n* = 3/19 patients did not improve) for down DVA and −140.97 ± 232.24 (*n* = 3/19 patients did not improve) for the up direction. Correlations were not done due to limited sample.

## Discussion

Our data suggest that improvements in patient reported subjective measures of dizziness and confidence, as well as fall risk are not explained by changes in the gain of the passively measured VOR. Furthermore, the improvements we report supersede the established MCID for the DHI, DGI, and gait speed (18, 26, 35). Recently, it has been reported that the VOR gain to passive head impulses improved 246% after completing a unique form of vestibular rehabilitation involving active ipsilesional head impulse rotation only (14). Lacour et al. also reveal limited change in the gain of the VOR in those patient groups that delayed their rehabilitation. One likely difference for the discrepancy between our data and that of Lacour et al. is the patient population. We studied a more complete lesion (deafferentation) relative to those of the Lacour study whom all had vestibular neuritis. Another explanation relates to the context of the gaze stability training. In our study, subjects performed gaze stability exercises using a sinusoid and lower frequency head rotation (<2 Hz) in yaw (and pitch). In contrast, the vHIT measures VOR gain during impulsive head rotation that includes higher frequency content of motion. We have recently shown that motor learning in the VOR is frequency specific, with evidence that VOR gain adaptation in the higher frequencies does not occur after lower frequency training (38). Thus, our results of VOR gain not changing after sinusoid gaze stability exercise implies that higher-frequency head movements are required during training if the goal is to change the VOR gain to higher frequencies. It remains possible that the absence of VOR gain change after our VPT program was related to the difference in training (active head rotation) vs. testing (passive head rotation). This is unlikely however, given recent evidence that VOR gain training using active impulses is adequate at improving the passive VOR (39).

In contrast to VOR gain, we did show the velocity of the overt compensatory saccades (CS) did reduce during ipsilesional head impulses after 5 weeks of VPT (albeit other CS metrics did not). Prior studies have shown that the frequency and velocity of CS do change over time with VPT (13, 14, 40–42). Together, these data suggest that the current standard of care prescribing gaze stability exercises for VPT (sinusoidal head rotation) may not be restoring slow phase (i.e., vestibular) eye velocity during passive head rotation, but instead lead to an altered CS velocity putatively to improve gaze stability. Recent case study evidence suggests that improving the gain of the VOR to passive rapid head rotation using active bilateral impulse training is not only possible in vestibular hypofunction but also leads to improved gait and dynamic visual acuity (43, 44).

### Change in DVA With VPT

Although 79% of our patients did show improved DVA after VPT, a few of our patients with UVD did not show a significant change (*n* = 4/19 patients did not improve their ipsilesional DVA). This is in contrast to prior studies and one possible explanation for this discrepancy may be related to the different methods used to measure DVA (8, 10, 40). Our method of DVA testing tasked patients to identify 10 optotype choices, which should be more difficult to complete (23). Additionally, our version of the DVA test does not limit a maximum head velocity threshold. Prior versions of the computerized DVA test limit the optotype presentations to four (letter E oriented in up/down/left/right) and have set the upper head velocity threshold to 180 d/s. A second reason is that all of our patients had a more complete lesion given surgical excision of the vestibular schwannoma, where other studies examined unilateral vestibular hypofunction for broader reasons (i.e., neuritis). The fact that our group results were not significant is also in-part related to the large variability in LogMAR scores, with some individuals doing much worse on their post testing measure. We investigated the surgical record of the four subjects in our study who did not show improvement in DVA. In summary, three of the four patients had facial paralysis and indication of central brainstem or cerebellar changes as evidence by statements including “small acute/subacute infarction postero-inferiorly in the right cerebellar hemisphere, not directly at the site of recent surgery”; “stable degree of mass effect on the left brachium pontis”; and “patchy edema within the dorsal and dorsolateral aspects of the right cerebellar hemisphere.” Therefore, it remains possible that the absent change in DVA from these three patients is related to their central pathology. We cannot explain why the fourth subject showed no change in DVA. Finally, it remains possible that patients improve their DVA via strategies different from the mechanisms we measured. For example, the unique roles of sensory re-weighting or cervical proprioception may also explain the change in DVA with vestibular rehabilitation.

### The Role of Otolith Function on Compensation From Vestibular Rehabilitation

Our data is the first behavioral evidence to show correlations between improved DVA (putative semicircular canal function) and preserved otolith function (smaller magnitude asymmetry ratio) as measured by ocular and cervical VEMP. It has recently been shown that labyrinthectomized mice also missing otolith function (otopetrin 1), are unable to adapt their angular VOR gain as well as healthy mice (45). Our data support the murine evidence that otolith function does appear to have a critical role in compensation to semicircular canal damage. Additional and recent evidence also suggests that the otolith pathways as measured via the head tilt and head/trunk tilt tests improve more quickly than the semicircular canal pathways in patients recovering from vestibular schwannoma resection (46, 47).

### Limitations

We lost a significant number of subjects to follow up based on being a tertiary care facility that draws patients from distances inconvenient for return visits. Additionally, given we did not include a patient control group, we are unable to know for sure whether rehabilitation was the reason why people subjectively and objectively improve as we report. Furthermore, we are unable to determine if our intervention may have led to changes in vestibular physiological measures were the patients examined closer to their surgical onset—although we did select “chronic patients” to avoid a possible confound of natural recovery. The exercise categories we developed in attempt to standardize the rehabilitation provided have not been validated, though do represent the current standard of care given rehabilitation providers commonly choose exercise difficulty based on clinical presentation. Finally, a greater sample size is needed to establish the unique roles that residual semicircular and otolith function have on improving impairments in patients with vestibular hypofunction.

## Conclusion

After 5 weeks of vestibular rehabilitation, subjective and performance outcomes were clinically and statistically improved despite absent change in VOR gain. Some individuals did have evidence for physiologic change. Dynamic visual acuity improved in 79% of our subjects, and was correlated with otolith, not semicircular canal function.

## Data Availability Statement

The datasets generated for this study are available on request to the corresponding author.

## Ethics Statement

The studies involving human participants were reviewed and approved by Johns Hopkins Internal Review Board. The patients/participants provided their written informed consent to participate in this study.

## Author Contributions

MS, JM, and YG: concept and data analysis. JM and YG: data collection. DR and MS: software development. JM, MS, YG, and DR: manuscript preparation.

### Conflict of Interest

The authors declare that the research was conducted in the absence of any commercial or financial relationships that could be construed as a potential conflict of interest.

## Supplementary Material

Data Sheet 1 Johns Hopkins University Vestibular Rehabilitation Exercise and Compliance Log.
