# Supplementary material for: Improvement After Vestibular Rehabilitation Not Explained by Improved Passive VOR Gain
Source: Front Neurol. 2020 Feb 20;11:79. doi: 10.3389/fneur.2020.00079 (PMC7044341; doi:10.3389/fneur.2020.00079)
Supplement: Data Sheet 1 — Johns Hopkins University Vestibular Rehabilitation Exercise and Compliance Log. [file Data_Sheet_1.pdf]

Johns Hopkins University
Vestibular Rehabilitation Exercise and Compliance Log

Week 1 Level A Home Exercise Program:

	Name:	Time	Sun	Mon	Tue	Wed	Thurs	Fri	Sat
	Date:	Minutes	Time	Time	Time	Time	Time	Time	Time
1	X 1 viewing in standing, horizontal direction, target arms length away, feet in a comfortable position	1.5							
2	X 1 viewing in standing, vertical direction, target arms length away, feet in a comfortable position	1.5							
3	Static stance in corner of room with feet positioned together, arms crossed	1.5							
4	Static stance in corner of room with feet together, looking up at the ceiling	1.5							
5	Walking (stepping) with feet close together, in a more narrowed base of support, arms crossed, looking straight ahead	1.5							
6	Walking with slow horizontal head movement (1 head turn every two steps), gazing to right and left with each head turn	1.5							
	Walk outside for exercise 10-15 minutes.								
	Total Time Duration								

Comments:

Copyright Michael C Schubert PT PhD FAPTA Laboratory of Vestibular NeuroAdaptation

Johns Hopkins University
Vestibular Rehabilitation Exercise and Compliance Log

Week 1 Level B Home Exercise Program:

	Name:	Time	Sun	Mon	Tue	Wed	Thurs	Fri	Sat
	Date:	Minutes	Time	Time	Time	Time	Time	Time	Time
1	X 1 viewing in standing, target arms length away, horizontal direction, semi overlapped foot position	1.5							
2	X 1 viewing in standing, target arms length away, vertical direction, semi overlapped foot position	1.5							
3	Static stance in corner of room with feet semi overlapped, arms crossed	1.5							
4	Static stance in corner of room with feet overlapped, arms crossed, looking up to the ceiling.	1.5							
5	Walking (stepping) with feet close together and overlapped, arms crossed, looking straight ahead	1.5							
6	Walking with horizontal head movement in a narrowed base of support, gazing to right and left with each head turn	1.5							
	Walk outside for exercise 10-15 minutes.								
	Total Time Duration								

Comments:

Copyright Michael C Schubert PT PhD FAPTA Laboratory of Vestibular NeuroAdaptation

Johns Hopkins University
Vestibular Rehabilitation Exercise and Compliance Log

Week 1 Level C Home Exercise Program:

	Name:	Time	Sun	Mon	Tue	Wed	Thurs	Fri	Sat
	Date:	Minutes	Time	Time	Time	Time	Time	Time	Time
1	X 1 viewing, horizontal direction, target arms length away, with one foot on ground, one foot on step	1.5							
2	X 1 viewing, vertical direction, target arms length away, with one foot on ground, one foot on step	1.5							
3	Static stance in corner of room with feet semi overlapped, arms crossed	1.5							
4	Semi tandem stance in corner of room with feet in semi overlapped position, head tilt up to the ceiling, standing on tip toes, arms crossed.	1.5							
5	Walking with feet semi overlapped, arms crossed, with deliberate horizontal head turns every 1-2 steps	1.5							
6	Walking with horizontal head movement every step while counting backwards from 100 by 3s	1.5							
	Walk outside for exercise 10-15 minutes.								
	Total Time duration								

Comments:

Copyright Michael C Schubert PT PhD FAPTA Laboratory of Vestibular NeuroAdaptation
